# Antiproliferative Effects of New Dimeric Ellagitannin from *Cornus* *alba* in Prostate Cancer Cells Including Apoptosis-Related S-Phase Arrest

**DOI:** 10.3390/molecules21020137

**Published:** 2016-01-23

**Authors:** Kwan Hee Park, Jun Yin, Ki Hoon Yoon, Yoon Jeong Hwang, Min Won Lee

**Affiliations:** Laboratory of Pharmacognosy and Natural Product based Medicine, College of Pharmacy, Chung-Ang University, Seoul 156-756, Korea; kwany1982@naver.com (K.H.P.); yinjun89@naver.com (J.Y.); ykhj33@naver.com (K.H.Y.); g_intention@naver.com (Y.J.H.)

**Keywords:** *Cornusalba*, hydrolysable tannins, ellagitannins, antioxidant, anti-inflammatory, anti-proliferation, prostate cancer

## Abstract

Activity-guided isolation of 80% acetone extract of *Cornus* *alba*, which is traditionally used as an anti-inflammatory, hemostatic and diuretic in Korea, yielded one novel compound, tentatively designated cornusiin H (**13**), together with 12 known compounds. The known compounds included four flavonoids (catechin (**1**), quercetin-3-*O*-β-d-glucuronide (**2**), quercetin-3-*O*-β-d-glucopyranoside (**3**), kaempferol-3-*O*-β-d-glucopyranoside (**4**)) and eight hydrolysable tannins (gallic acid (**5**), 2,6-di-*O-*galloyl-hamamelofuranoside (**6**), 2-galloyl-4-caffeoyl-l-threonic acid (**7**) 2,3-di-*O*-galloyl-4-caffeoyl-l-threonic acid (**8**), 1,2,3,4,6-penta-*O*-galloyl-β-d-glucopyranoside (**9**), cornusiin B (**10**), cornusiin A (**11**) and camptothin B (**12**)). All compounds exhibited potent 1,1-diphenyl-2-picrylhydrazyl (DPPH)-free radical scavenging activity. Especially, the radical scavenging activities of **6** and **9**–**13** were higher than that of vitamin C. Compounds **9**, **11**, **12** and **13** inhibited the production of nitric oxide (NO) in lipopolysaccharide-stimulated RAW264.7 cells to the same degree as *N*^G^-Monomethyl-l-arginine (l-NMMA). When the antiproliferative effects of the isolated compounds were assessed in prostate cancer cells, the dimeric ellagitannins (**11**–**13**) selectively inhibited LNCaP hormone-dependent prostate cancer cells. Flow cytometry analysis indicated that the dimeric ellagitannins induced apoptosis and S-phase arrest. These results suggest that dimeric ellagitannins from *Cornus* *alba* can be developed as functional materials or herbal medicines for prostate tumors such as benign prostate hyperplasia and early-stage prostate cancer.

## 1. Introduction

Prostate tumors can be categorized as benign prostate hyperplasia (BPH), which is an overgrowth of the prostate caused by extensive androgen-dependent tissue remodeling [1], and prostate cancer (PCa), which is a malignant tumor caused by oncogenic mutations, aberrant signaling or inflammatory conditions [2]. The proliferation of prostate tissue which could make urination difficult is an important problem for BPH [3]. The α_1_-Adrenergic agonists and 5α-reductase inhibitors are typically used to treat BPH, with the goals of improving urination and decreasing prostate growth. However, long-term issues with these medications include adverse effects, such as low blood pressure and sexual dysfunction. Hormonal therapy is used for early-stage prostate cancer. Frequently, the cancer becomes hormone-refractory. Being a typical chronic disease in middle-aged and old men, prostate tumors are likely to increase with age. BPH has been described in 62% of European middle-aged men [4]. PCa, the most common cancer in Western men, occurred mostly between 54 and 75 years of age [2]. The incidence of prostate tumors is rapidly increasing in Korea. A recent Korean survey reported that the prevalence of BPH was about 40% in men over 65 years of age [5], with the incidence of PCa increasing by an average of 12.1% annually over the past decade, ranking as the fifth most prevalent cancer in men [6]. Also, on the worldwide scale, PCa, as second in the estimated new cases and fifth in the estimated deaths in males, is one of the most prevalent cancers [7].

Plant extracts have been widely used as an alternative treatment of prostate tumors. *Serenoa* *repens* (Saw palmetto), *Hypoxis* *rooperi* (South African star grass), *Urtica* *dioica* (Stinging nettle), *Curcubita* *pepo* (Pumpkin seed oil), *Secale* *cereale* (Rye pollen), *Pygeum* *africanum* (African plum), alone and in various combinations, are commercially available for the management of BPH and lower urinary tract symptoms (LUTS) [8]. Recently, a Chinese herbal mixture designated prostate cancer-spes (PC-SPES) which is a mixture of eight different herbs, including *Chrysanthemum* *morifolium*, *Ganoderma* *lucidum*, *Glycyrrhiza* *glabra*, *Isatis* *indigotica*, *Panax* *pseudoginseng*, *Rabdosia* *rubescens*, *Scutellaria* *baicalensis* and *Serona* *repens*, became commercially available for the treatment of PCa [9]. Phytosterol (β-sitosterol and lupeol) and phytoestrogen (genistein) are major compounds obtained from plant extracts that reportedly inhibit 5α-reductase and synthesis of prostagladin, impose androgen and α_1_ blockades, and lessen inflammation.

The Cornus species of plants is also used as an East Asian folk remedy for urinary health. *C.* *officinalis* hips are a renowned natural tonic in aging men [10], and the stems of *C.* *walteri* are used as a diuretic [11,12]. Various phytochemicals including lignans, iridoids, terpenoids, flavonoids and tannins have been more recently isolated from these species [13,14,15], and Cornus extracts reportedly possess antioxidative, anti-inflammatory and anti-cancer biological activities [16]. *Cornus* *alba* (CA), also known as red-barked or Siberian dogwood, is native to Siberia, northern China and Korea [16]. The stems and leaves of CA have been used as antiphlogistic, hemostatic and diuretic treatments in Korea. [17] This ethnopharmacologic usage comes despite limited knowledge of chemistry and biological activity of this plant.

The present study was undertaken to provide clarity on its phytochemicals and biological activities. We tried to isolate active constituents and evaluated their biological activities with the goal of developing natural prostate tumor medications.

## 2. Results and Discussion

### 2.1. Structural Identification and Elucidation

**Figure 1:**
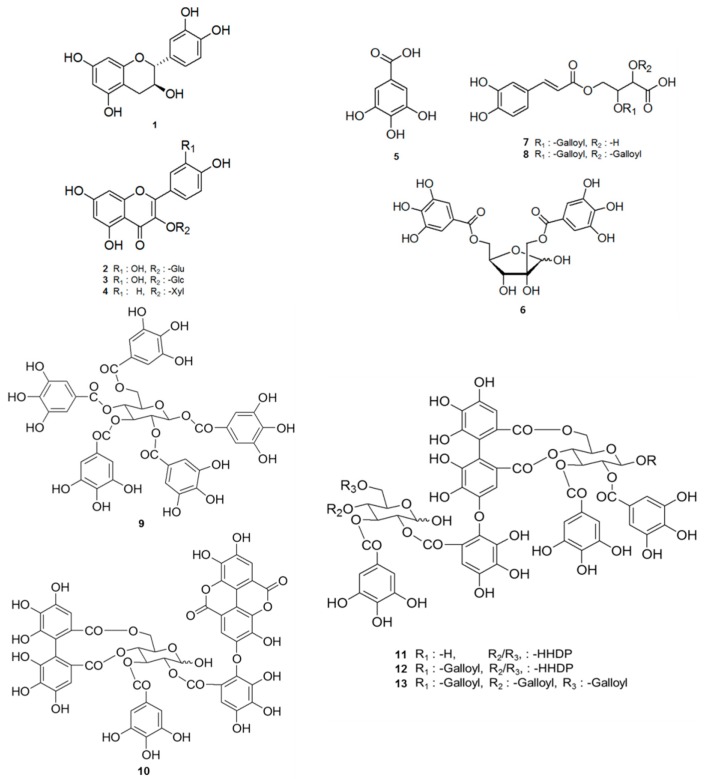
The structures of compounds **1**–**13** isolated from CA.

Twelve known compounds were identified as catechin (**1**) [18], quercetin-3-*O*-β*-*d-glucuronide (**2**) [19], quercetin-3-*O*-β*-*d-glucopyranoside (**3**) [19], kaempferol-3-*O*-β*-*d-glucopyranoside (**4**) [20], gallic acid (**5**) [18], 2,6-di-*O*-galloyl-hamamelofuranoside (**6**) [21], 2-galloyl-4-caffeoyl-l-threonic acid (**7**) [22], 2,3-di-*O*-galloyl-4-caffeoyl-l-threonic acid (**8**) [22], 1,2,3,4,6-penta-*O*-galloyl-β-d-glucopyranoside (**9**) [23], cornusiin B (**10**) [24], cornusiin A (**11**) [25] and camptothin B (**12**) [24,25]. The spectroscopic data of compounds **1**–**12** were carefully compared with values reported in the literature (Figure 1).

Compound **13** was obtained as an amorphous brown powder. It was detected as a dark blue spot by spraying with FeCl_3_ solution, and as a yellow spot by spraying with H_2_SO_4_ solution, followed by heating on TLC. The molecular formula was C_75_H_56_O_48_ as indicated by HR-FAB-MS data (*m*/*z* 1723.1865 [M − H]^−^ calculated for C_75_H_55_O_48_, 1723.1863). On ^1^H-NMR, all signals were duplicated, and one pair of downfield-shifted anomeric proton signals were observed at δ 6.18, 6.18 (1.7 H in total, each d, *J* = 8.4 Hz, H-1_R_ of each two forms). (Figure S3) These findings suggested that compound **13** should exist as an equilibrium mixture of two tautomers (α_L_-β_R_:β_L_-β_R_ = 1:0.7) with an acylated anomeric center of the glucose core. Careful analysis of the ^1^H-NMR spectrum revealed six galloyl groups and one valoneoyl (val) group (δ 7.00, 7.00, 7.01, 7.03, 7.06, 7.07, 7.10, 7.11, 7.12, 7.16 and 7.17 (each s, 5 × galloyl H-2 and 6; val H_C_), 6.93 and 6.86 (each s, galloyl H-2 and H-6), 6.69 and 6.68 (each s, val H_A_) and 6.19 and 6.19 (each s, val H_B_)) in the aromatic region, together with two glucose cores with a ^4^C_1_ conformation (δ 3.80–6.23 (large coupling constants in all sugar protons)) in the sugar region. (Figures S2 and S3)

The positive cotton effect at the short wavelength region ([θ]_224_ 8.81) in the CD spectrum indicated that the absolute configuration of the valoneoyl group was *S* [26]. The downfield-shifted H-4_R_ (δ_H_ 5.11, 5.12) as well as the separately observed H-6a_R_ (δ_H_ 5.27–5.32) and H-6b (δ_H_ 3.76–3.96), which were correlated with acylated anomeric H-1_R_ (δ_H_ 6.18, 6.18) in TOCSY, suggested that the valoneoyl group should be connected at OH-4/OH-6 of the right glucose core [27]. (Figure S4) In the left glucose core, the downfield-shifted H-4_L_ (δ_H_ 5.50, 5.57) as well as the adjoined H-6a_L_ (δ_H_ 4.47, 4.51) and H-6b_L_ (δ_H_ 4.23, 4.30), which correlated with the non-acylated H-1_L_ (δ_H_ 4.74, 5.42) in TOCSY, (Figure S4) and no observation of any characteristic chemical shifts of the HHDP group at δ 6.40–6.60 indicated that the two galloyl groups should connect with the OH-4 and OH-6 of the left glucose core, respectively. (Figure S3) The upfield-shifted H-1 of the β-anomer (δ 4.74 (0.7H, *J* = 7.8 Hz, H-1_L_ of β*-*β form) compared with the α-anomer in the left-side glucose core (δ 5.42 (1H, d, *J* = 3.0 Hz, H-1_L_ of α*-*β form)) indicated that the valoneoyl group at OH-2 of the left-side glucose core induced an anisotropic shielding effect in H-1_L_. (Figure S1) In addition, the downfield-shifted Val H_A_ at δ_H_ 6.68 and 6.69, due to absence of the shielding effect by the galloyl group at OH-3 in the right glucose core, indicated that the left glucose core is connected at the bottom of the valoneoyl group. The ellagitannin composed with an unacylated anomeric center and substituted by two galloyl groups at OH-4 and OH-6 in the left glucose core was tentatively designated cornusiin H.

### 2.2. 1,1-Diphenyl-2-picrylhydrazyl (DPPH) Radical Scavenging Activity

Reducing oxidative stress is related to the anti-cancer and anti-inflammatory effect. [28] To assess the antioxidative activity of the compounds isolated from CA, DPPH radical scavenging activity was investigated. All compounds showed radical scavenging activity in a dose-dependent manner (data not shown). Especially compounds **6** and **9**–**13**, which are hydrolysable tannins, possessed more potent DPPH free radical scavenging activities than the vitamin C positive control, while the scavenging activities of simple gallic acids (**5**) and galloyl caffeoyl threonic acids (**7**, **8**) were moderate (Table 1). The hydrolysable tannins (**6** and **9**–**13**) had more than two galloyl groups known to eliminate free radicals, and their efficiency depended on the number of galloyl groups (**6** < **9** < **10**, **11** < **12**, **13**). Flavonols are well-known antioxidative compounds. Especially, the hydroxyl group at C-5, the ketone at C-4 and B-ring phenolic hydroxyl groups are crucial as electron acceptors. In this case, the radical scavenging activities of compounds **2** and **3**, which have a 3′,4′-dihydroxylphenyl B-ring, were more potent than compound **4**, which possesses a 4′-hydroxylphenyl B-ring.

**Table 1:** IC_50_ values of compounds **1**–**13** against scavenging DPPH radicals or inhibition of NO production on LPS-stimulated RAW264.7 cells.

Samples	IC_50_ (μM) ^a^	
	DPPH Radical Scavenging Activity	NO Production Inhibitory Activity
**1**	31.17 ± 0.31	100<
**2**	23.64 ± 1.26	100<
**3**	24.08 ± 0.90	100<
**4**	66.45 ± 5.79	100<
**5**	31.71 ± 1.42	100<
**6**	9.47 ± 0.42	100<
**7**	28.39 ± 1.68	100<
**8**	20.98 ± 1.10	100<
**9**	8.45 ± 1.99	28.59 ± 0.82
**10**	6.72 ± 0.16	98.74 ± 8.91
**11**	6.01 ± 0.16	20.99 ± 0.14
**12**	5.40 ± 0.16	20.93 ± 0.15
**13**	5.52 ± 0.28	21.26 ± 0.38
Vit.C ^b^	13.30 ± 0.31	-
l-NMMA ^b^	-	17.10 ± 0.25

^a^ Values are presented as the mean ± SD (*n* = 3); ^b^ Positive controls. Vit.C: l-ascorbic acid; l-NMMA: *N*^G^-monomethyl-l-arginine.

### 2.3. Inhibition of Nitric Oxide (NO) Production

It is reported that there is a strong correlation between the inflammation and the pre-cancerous lesion, [29] so the anti-inflammatory effect could also partly reflect the anti-cancer effect. To evaluate the anti-inflammatory activities of the compounds isolated from CA, their inhibition of NO production in LPS-stimulated RAW264.7 cells was investigated as described previously [30]. While the flavonoids (**1**–**4**) and mono- or di-galloyl substituted tannins (**5**–**8**) had no effect, the hydrolysable tannins (**9**–**13**) dramatically inhibited NO production to almost the same degree as *N*^G^-monomethyl-l-arginine (l-NMMA), except for **10**, which has a substituted lactonized valoneoyl group (Table 1). These findings indicated that the lactonized valoneoyl moiety may not be helpful for the intracellular effects of hydrolysable tannins.

### 2.4. Antiproliferative Activity on Prostate Cancer Cells

**Figure 2:**
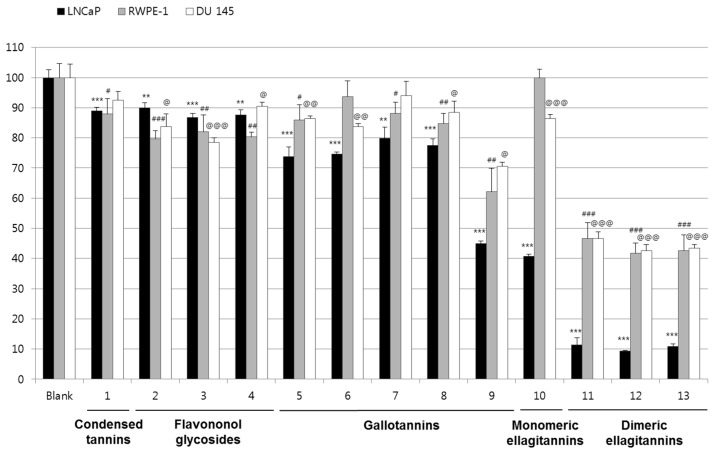
The antiproliferative effects of **1**–**13** from *C. alba*. Each compound was treated at the concentration of 50 μM into androgen-dependent prostate tumor LNCaP, androgen-independent prostate tumor DU145 and normal prostate epithelial RWPE-1 cells. The results were expressed as mean ± S.D. of triplicated experiments. **: *p* < 0.01, ***: *p* < 0.001, compare with LNCaP Blank group; #: *p* < 0.05, ##: *p* < 0.01, ###: *p* < 0.001, compare with RWPE-1 Blank group; @: *p* < 0.05, @@: *p* < 0.01, @@@: *p* < 0.001, compare with LNCaP Blank group.

Besides the indirect anti-cancer effect, which includes anti-oxidative and anti-inflammatory activities, the antiproliferative activity on cancer cells could more directly affect the anti-cancer activity. To evaluate the antitumor effects of the compounds isolated from CA, the antiproliferative effect of each compound was determined using a 3-(4,5-Dimethylthiazol-2-yl)-2,5-Diphenyltetrazolium Bromide (MTT) assay on androgen-dependent LNCaP and androgen-insensitive DU145 cell lines. The antiproliferative activity of 50 μM of each compound was screened in LNCaP and DU145 cells and RWPE-1 normal prostate epithelial cells together with other tannin-related compounds previously isolated from other plant sources. The compounds were divided into five groups as follows: (1) condensed tannins: **1**; (2) flavononol glycosides: **2**–**4**; (3) gallotannins: **5**–**9**; (4) monomeric ellagitannins: **10**; and (5) dimeric ellagitannins: **11**–**13**. All dimeric ellagitannins showed selective antiproliferative effects on LNCaP cells. These compounds decreased the cell viability of LNCaP four to five times more than that of RWPE-1. This finding suggested that the HHDP group on ellagitannins might increase the antiproliferative effects of androgen-dependent LNCaP. Interestingly, compound **10**, which possesses a lactonized valoneoyl group at OH-2, was decreased. Thus, the rigid valoneoyl group at OH-2 and the flexible galloyl group at OH-2 and OH-3 may be essential for selective antiproliferation. The antiproliferative effects of compounds **9**–**13** were assessed at various concentrations using LNCaP and DU145 cells. Especially, dimeric ellagitannins (**11**–**13**) inhibited the proliferation of LNCaP cells seven times more potently than DU145 cells, which means dimeric ellagitannins could inhibit the proliferation of hormone-dependent PCa cells more powerfully than hormone-insensitive PCa cells (Table 2), and decreased the cell viability of LNCaP cells more effectively than monomeric ellagitannins did (Figure 2).

**Table 2:** IC_50_ values of **9**–**13** against the cell viability of LNCaP and DU145.

Samples	IC_50_ (μM) ^a^	
	LNCaP	DU145
**9**	50<	100<
**10**	50<	100<
**11**	6.31 ± 0.23	48.32 ± 5.12
**12**	6.03 ± 0.16	41.48 ± 2.42
**13**	5.97 ± 0.15	44.06 ± 1.43
EGCG ^b^	50<	100<

^a^ Values are presented as the mean ± SD (*n* = 3); ^b^ Positive controls.

### 2.5. Induction of Cell Cycle Arrest and Apoptosis

**Figure 3:**
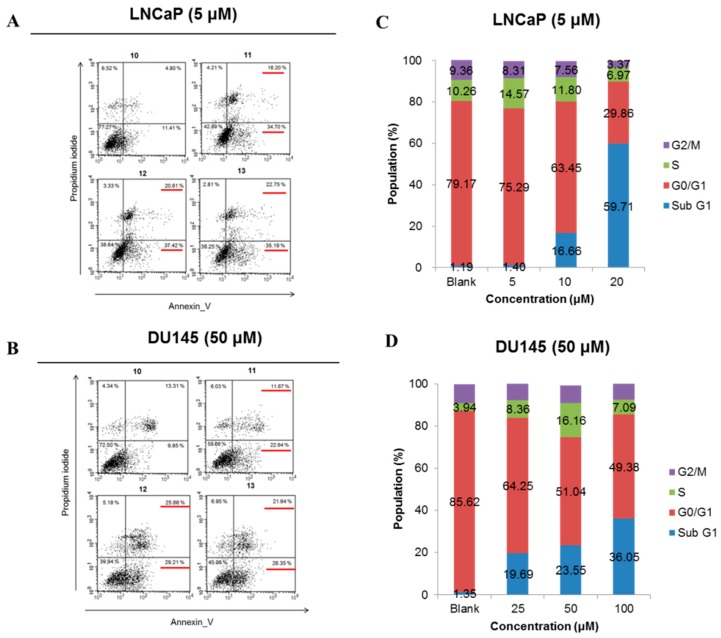
Induction of apoptosis (**A**,**B**) and cell cycle arrest (**C**,**D**) by dimeric ellagitannins (**11**–**13**) from *C.* *alba*. The apoptosis induction and cell cycle distribution were analyzed on flow cytometry. Concentrations of 5 or 50 μM of each compound were used to treat LNCaP and DU145 cells for 48 h, respectively. In A and B, the percentages of cells in each quadrant are indicated as follows—lower left: normal, lower right: early apoptosis, upper right: late apoptosis, upper left: necrosis.

The apoptosis effect of PCa is helpful to assay the anti-cancer effect. To observe cell cycle distribution and apoptosis by the ellagitannins (**9**–**13**) obtained from CA, flow cytometry analysis was performed. Compounds **11**–**13** which effectively are dimeric ellagitannins induced apoptosis within 48 h, and the potency of dimeric ellagitannins was two to three times better than that of monomeric ellagitannins (Figure 3). Compound **11** elevated the subG1 phase of both LNCaP and DU145 cells in a dose-dependent manner, and the potency for LNCaP cells was 10 times greater than that of DU145 cells (Figure 3). Compound **11** applied at a low concentration also increased the S-phase, while the cells in G0/G1 and G2/M were decreased (Figure 3). These findings suggested that dimeric ellagitannins may induce S-phase arrest and apoptosis.

## 3. Materials & Methods

### 3.1. Plant Material

CA (5.7 kg) was collected at the Korea National Arboretum (Pocheon, Korea) in September 2011. The identification of the material was certificated by Mr. Kim Sung Sik, Curator in the Department of Horticulture and Landscape Design. A voucher specimen (CA2011) was deposited at the herbarium of the College of Pharmacy, Chung-Ang University (Seoul, Korea).

### 3.2. General Procedures

Column chromatography was performed using a Sephadex LH-20 column (10–25 μm; GE Healthcare Bio-Science AB, Uppsala, Sweden) or MCI CHP 20P column (75–150 μm; Mitsubishi Chemical, Tokyo, Japan). Daisogel (40–60 μm; Daiso, Osaka, Japan) and Toyopearl HW-40F (30–60 μm; Tosoh Corp., Tokyo, Japan) were used in the stationary phase in a middle-pressure liquid chromatography (MPLC) system (Gilson, Seoul, Korea). For monitoring of each fraction, thin layer chromatography (TLC) was carried out using pre-coated silica gel 60 F_254_ plates (Merck, Darmstadt, Germany) that were developed with chloroform, methanol and water (6:4:1 volume ratio), or benzene, ethylformate and formic acid (1:7:1 or 1:7:2 volume ratio). The spots were detected using ultraviolet radiation (254 nm) and by spraying with a FeCl_3_ solution and 10% H_2_SO_4_ followed by heating. Structural identification was by nuclear magnetic resonance (NMR; Varian, Palo Alto, CA, USA), high resolution fast atom bombardment mass spectrum (HRFAB-MS; JEOL, Tokyo, Japan) and circular dichroism (CD; Jasco, Tokyo, Japan).

### 3.3. Extraction and Isolation

Several extractions of CA (5.7 kg) using 80% acetone at room temperature followed by removal of the acetone under vacuum yielded 463 g of extract. The extract was dissolved in water and filtered through Celite 545 (Duksan Pure Chemical, Ansan, Korea). Then 356 g of water-soluble fraction was obtained together with 89 g of water-insoluble residue; 243 g of the water-soluble fraction applied to a Sephadex LH-20 column (15 × 100 cm) equilibrated with water. The column was eluted with a water-methanol gradient system and washed in 60% acetone, which yielded 14 fractions.

Fraction 6 (2.5 g) was applied to Daisogel (3 × 50 cm, here and hereafter) with the water-methanol gradient in the MPLC system (5 mL/min, 280 nm, here and hereafter) to yield quercetin-3-*O*-β-d-glucuronide (**2**, 143 mg). Fraction 7 (2.7 g) was applied to Daisogel with the water-methanol gradient in the MPLC system (5 mL/min, 280 nm, here and hereafter) to yield gallic acid (**5**, 121 mg). Fraction 8 (12.8 g) was applied to a MCI CHP20P column (5 × 60 cm, here and hereafter) with the water-methanol gradient to obtain catechin (**1**, 27 mg), quercetin-3-*O*-β-d-glucopyranoside (**3**, 157 mg), kaempferol-3-*O*-β-d-glucopyranoside (**4**, 24 mg), hamamelitannin (**6**, 1.2 g) and 2-galloyl-4-caffeoyl-l-threonic acid (**7**, 32 mg). Fraction 11 (3.4 g) was repeatedly applied to a Daisogel column using the water-methanol gradient in the MPLC system to obtain 2,3-di-galloyl-4-caffeoyl-l-threonic acid (**8**, 27 mg). Fraction 13 (**31**, 6 g) was applied to a MCI CHP20P column with the water-methanol gradient to obtain five sub-fractions. Fraction 13-2 was applied to a Daisogel column with the water to 20% methanol gradient in the MPLC system and was further separated by column chromatography on a Sephadex LH-20 column with a water-methanol-60% acetone gradient system to obtain cornusiin B (**10**, 140 mg) and cornusiin A (**11**, 1.2 g). Fraction 13-3 was applied to a Sephadex LH-20 column (2.5 × 50 cm, here and hereafter) with the water-methanol-60% acetone gradient system and further separated by column chromatography on a MCI CHP20P column with water-methanol gradient system resulted to three additional sub-fractions. Fr 13-3-1 was chromatographed by Toyopearl HW-40 (2.5 × 50 cm) with 70% methanol–70% acetone (10:0→7:3), and then yielded camptothin B (**12**, 815 mg). Fr 13-3-3 was chromatographed using a Sephadex LH-20 column with a 100% ethanol-100% methanol gradient system to obtain the novel compound tentatively designated cornusiin H (**13**, 312 mg). Fraction 13-4 was applied to Daisogel with the water-methanol gradient in a MPLC system to yield 1,2,3,4,6-penta-*O*-galloyl-β-d glucopyranoside (**9**, 27 mg). TLC was developed using chloroform, methanol and water (6:4:1 volume ratio) and each spot was detected by spraying with 10% H_2_SO_4_ followed by heating and with ethanolic FeCl_3_. In the corresponding photograph, TLC patterns of CA 80% acetone extract and water-soluble/insoluble layer are shown in panel A and each sub-fraction obtained by column chromatography with Sephadex LH-20 from the water-soluble layer are shown in panel B.

#### Cornusiin H (**13**)

The product was an amorphous brown powder. Structural data are as follows:

HRFAB-MS *m/z*: 1723.1865 [M − H]^−^ (calculated for C_75_H_55_O_48_, 1723,1863)

CD (MeOH): [θ]_224_ 8.81, [θ]_258_ −0.89, [θ]_278_ 2.09

^1^H-NMR (600 MHz, Acetone-*d*_6_ + D_2_O): δ 3.88–3.92 (1.7H in total, each br d, *J* = 13.2, H-6b_R_ of each two form), 4.17 (0.7H, ddd, *J* = 2.4, 4.2, 10.2 Hz, H-5_L_ of β-β form), 4.23 (0.7H, dd, *J* = 4.2, 13.2 Hz, H-6b_L_ of β-β form), 4.30 (1H, dd, *J* = 4.2, 13.2 Hz, H-6b_L_ of α-β form), 4.47 (1H, dd, *J* = 4.2, 13.2 Hz, H-6a_L_ of α-β form), 4.48 (1H, m, H-5_R_ of α-β form), 4.51 (0.7H, dd, *J* = 4.2, 13.2 Hz, H-6a_L_ of β-β form), 4.53 (0.7H, m, H-5_R_ of β-β form), 4.17 (1H, ddd, *J* = 2.4, 4.2, 10.2 Hz, H-5_L_ of α-β form), 4.74 (0.7H, *J* = 7.8 Hz, H-1_L_ of β-β form), 5.11, 5.12 (1.7H in total, each t, *J* = 9.6 Hz, H-4_R_, of each two form), 5.13 (1H, dd, *J* = 3.0, 9.6 Hz, H-2_L_ of α-β form), 5.18 (0.7H, dd, *J* = 7.8, 9.6 Hz, H-2_L_ of β-β form), 5.27-5.32 (1.7H in total, each dd, *J* = 6.6, 13.2, H-6a_R_ of each two form), 5.42 (1H, d, *J* = 3.0 Hz, H-1_L_ of α-β form), 5.50 (0.7H, t, *J* = 9.6 Hz, H-4_L_ of β-β form), 5.55 (0.7H, dd, *J* = 7.8, 9.6 Hz, H-2_R_ of α-β form), 5.57 (1.0H, dd, *J* = 7.8, 9.6 Hz, H-2_R_ of β-β form), 5.57 (1H, t, *J* = 9.6 Hz, H-4_L_ of α-β form), 5.61 (0.7H, t, *J* = 9.6 Hz, H-3_R_ of β-β form), 5.62 (1.0H, t, *J* = 9.6 Hz, H-3_R_ of α-β form), 5.68 (0.7H, t, *J* = 9.6 Hz, H-3_L_ of β-β form), 6.06 (1H, t, *J* = 9.6 Hz, H-3_L_ of α-β form), 6.18, 6.18 (1.7H in total, each d, *J* = 8.4 Hz, H-1_R_ of each two forms), 6.19, 6.19 (1.7H in total, each s, val H_B_ of each two forms), 6.68 (1H, s, val H_A_ of α-β form), 6.69 (0.7H, s, val H_A_ of β-β form), 6.86 (0.7H, s, galloyl H-2, 6 of β-β form) and 6.93 (1H, s, galloyl H-2, 6 of α-β form), 7.00, 7.00, 7.01, 7.03, 7.06, 7.07, 7.10, 7.11, 7.12, 7.16, 7.17 (each s, 5 × galloyl H-2, 6 ; val H_C_ of each two forms).

^13^C-NMR (150 MHz, Acetone-*d*_6_ + D_2_O) : δ 62.2, 63.1 (C-6_L_ and C-6_R_ of each two forms), 67.5, (C-5_L_ of α-β form), 68.9 (C-4_L_ of each two forms), 70.1–70.4 (C-4_R_ of each two forms; C-3_L_ of α-β form) 70.9 (C-5_L_ of β-β form; C-5_R_ of each two forms), 71.9–72.2 (C-2_L_ and C-2_R_ of each two forms; C-3_R_ of α-β form, C-3_R_ of β-β form, C-3_L_ of β-β form), 89.9 (C-1_L_ of α-β form), 92.7 (C-1_R_ of α-β form, C-1_R_ of β-β form), 94.8 (C-1_L_ of β-β form), 104.0-104.5 (val C-3′), 107.1-107.3 (val C-3), 108.9-109.5 (gal C-2, gal C-6; val C-6′′), 112.6, 113.6, 114.9, 115.3, 116.6, 116.8 (val C-1, val C-1′, val C-1′′), 118.6-120.4 (gal C-1), 124.7, 125.0 (val C-2, val C-2′), 133.5-136.8 (val C-5, val C-5′, val-2′′), 138.1-139.0 (gal C-4), 139.0–139.7 (val C-3′′, val C-4′′), 142.3-142.5 (val C-5′′), 143.7-145.3 (gal C-3, gal C-5; val C-4, val C-6, val C-6′), 145.7-146.7 (val C-4′) and163.9-167.8 (gal C-7; val C-7, val C-7′ val C-7′′).

### 3.4. Measurement of DPPH Radical Scavenging Activity

Antioxidant activity was determined on the basis of the scavenging activity of the stable DPPH free radical (Sigma-Aldrich, St. Louis, MO, USA). Each 20 μL of sample in absolute ethanol was added to 180 μL of 0.1 mM DPPH in absolute ethanol. After mixing gently and standing for 30 min, the optical density was measured at 540 nm using an ELISA reader (TECAN, Salzburg, Austria). The free radical scavenging activity was calculated as inhibition rate (%) = [1 − (sample *O.D.*/control *O.D.*)] × 100; IC_50_ was the concentration that could scavenge 50% DPPH free radical. L-ascorbic acid was used as positive control.

### 3.5. Cell Culture

RAW 246.7, LNCaP and DU145 cells were purchased from the Korean Cell Line Bank. These cells were grown at 37 °C in a humidified atmosphere (5% CO_2_) in DMEM or RPMI (Sigma-Aldrich) containing 10% fetal bovine serum (FBS) and 100 IU/mL penicillin G (Gibco BRL, Grand Island, NY, USA).

### 3.6. Viability Assay

Approximately 10^5^/well of RAW264.7 or DU145 cells were seeded in wells of a 24-well plate and incubated for 4 h in 5% CO_2_ at 37 °C. LNCaP cells were seeded at a density of 10^4^ cells/well and were incubated for 24 h. The medium was replaced with phosphate buffered saline (PBS) containing 0.5 mg/mL of 3-(4,5-dimethylthiazol-2-yl)-2,5-diphenyltetrazolium bromide (MTT), and incubated for 4 h. The supernatant was removed and the MTT-formazan was dissolved in 200 μL dimethylsulfoxide. The extent of the reduction of MTT to formazan within the cells was measured at 540 nm with microplate reader (TECAN, Salzburg, Austria). The cell viability was calculated as sample *O.D.*/blank *O.D.*× 100 (%).

### 3.7. Measurement of Inhibition of Nitric Oxide (NO) Production

RAW 264.7 cells were cultured in wells of a 24-well plate and incubated for 4 h at 37 °C in a humidified atmosphere of 5% CO_2_. The cells were treated with i1 μg/mL lipopolysaccharide (LPS; Sigma-Aldrich) and incubated for 24 h. The NO content was determined by the Griess assay. Griess reagent (100 μL of 0.1% naphthylethylenediamine and 1% sulfanilamide in 5% H_3_PO_4_ solution; Sigma-Aldrich) was added to 100 μL of each supernatant. NO was then measured at 540 nm with a microplate reader (TECAN). NO was quantified using a sodium nitrite standard curve as previously described [30]. NO inhibitory activity was calculated as [1 − (sample *O.D.* − blank *O.D.*)]/( negative control *O.D.* − blank *O.D.*) × 100 (%).

### 3.8. Flow Cytometry Analysis of Cell Cycle Arrest

The cells were harvested and washed once in PBS. The cell pellet was re-suspended in 400 μL PBS containing 2% FBS and fixed in 1.2 mL 70% ethanol for 24h at −20 °C. Cells were washed twice in PBS and treated with 0.1% Triton X-100 and ribonuclease A (100 μg/mL) in PBS for 20 min. Propidium iodide (PI; 25 μg/mL) was added and flow cytometry was carried out using a BD-LSR II flow cell cytometer (BD, San Jose, CA, USA) using Cell Quest software.

### 3.9. Flow Cytometry Quantification of Apoptosis

Cells were harvested and washed once in PBS and once in annexin V binding buffer. The cells were resuspended in binding buffer and stained with annexin V-fluorescein isothiocynate (FITC) and PI for 15 min in the dark at 4 °C. The fluorescence was analyzed by flow cytometry as described above. The percentages of necrotic cells, early and late apoptotic cells, and viable cells were compared.

## 4. Conclusions

A new dimeric ellagitannin, designated cornusiin H (**13**), was isolated from CA together with 12 known phenolic compounds. Dimeric ellagitannins (**11**–**13**) showed potent DPPH radical scavenging activity and inhibited NO production in LPS-stimulated RAW 264.7 cells. Also, they selectively inhibited proliferation of LNCaP hormone-dependent prostate tumor cells. The rigid 4,6-HHDP group and flexible 2,3-*O*-digalloyl were essential for the antiproliferative effects and dimerization via the valoneoyl group’s increased activity. The dimeric ellagitannins efficiently induced apoptosis as well as S-phase arrest. Thus, the dimeric ellagitannins or ellagitannin-rich fraction from CA might be developed as functional material to alleviate prostate tumors (BPH or early-stage PCa) through future clinical study.

## Supplementary Materials

Supplementary materials can be accessed at: http://www.mdpi.com/1420-3049/21/2/137/s1.
