# Supplementary material for: Antiproliferative Effects of New Dimeric Ellagitannin from Cornus alba in Prostate Cancer Cells Including Apoptosis-Related S-Phase Arrest
Source: Molecules. 2016 Jan 23;21(2):137. doi: 10.3390/molecules21020137 (PMC6273526; doi:10.3390/molecules21020137)
Supplement: Supplementary file 1 [file molecules-21-00137-s001.pdf]

Supplementary Materials: Antiproliferative Effects of New Dimeric Ellagitannin from *Cornus alba* in Prostate Cancer Cells Including Apoptosis-Related S-Phase Arrest

Kwan Hee Park †, Jun Yin †, Ki Hoon Yoon, Yoon Jeong Hwang and Min Won Lee *

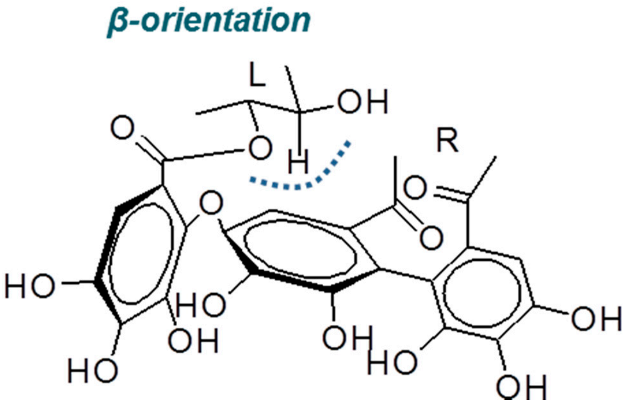

Figure S1. Partial structure of the *β*-anomer of compounds **11**, **12** and **13**.

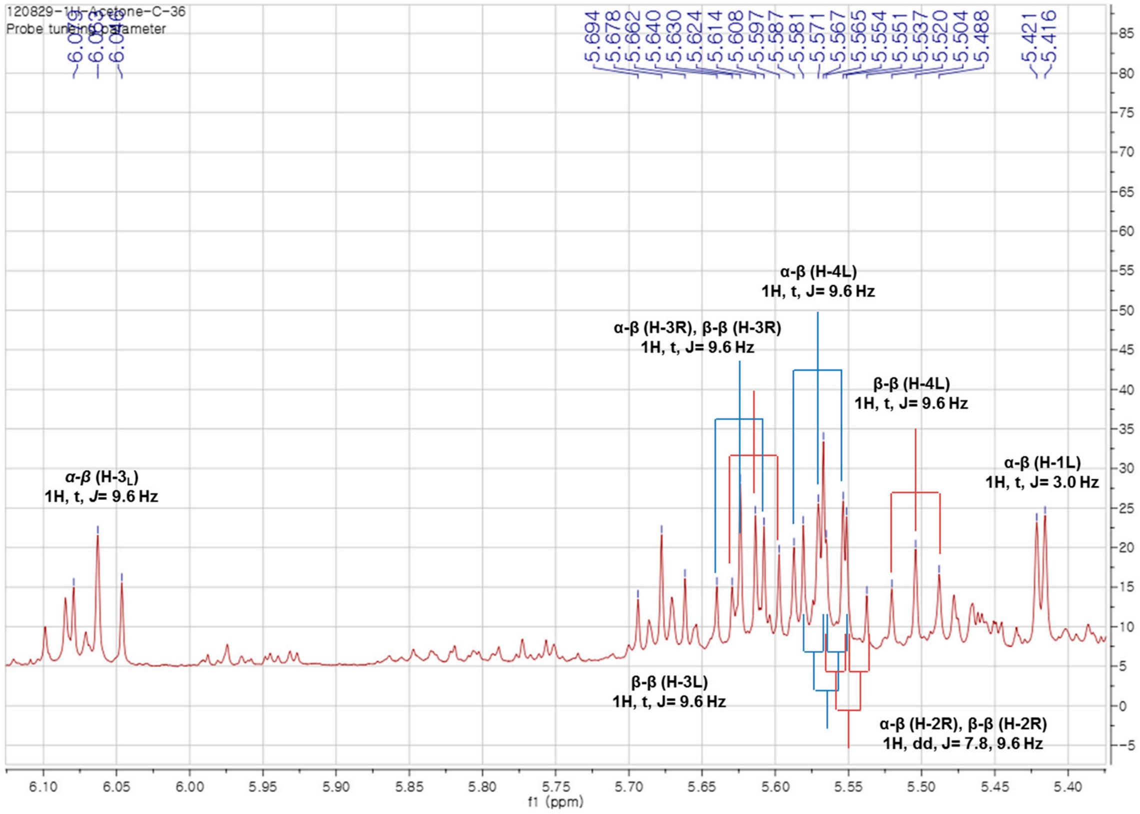

Figure S2. *Cont.*

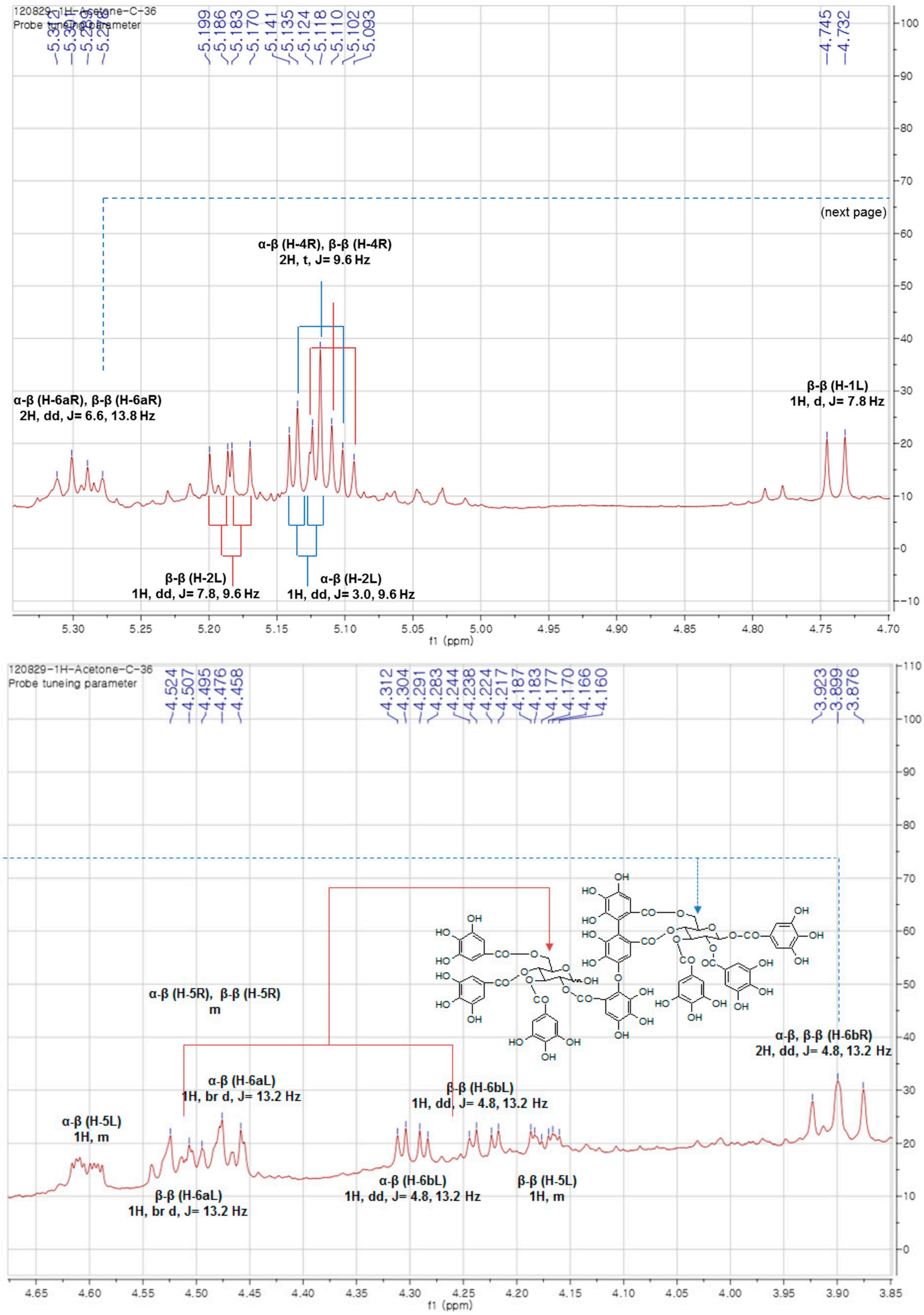

Figure S2. Two glucose cores with a $^4C_1$ conformation [δ 3.80–6.23 (large coupling constants in all sugar protons)] in sugar region.

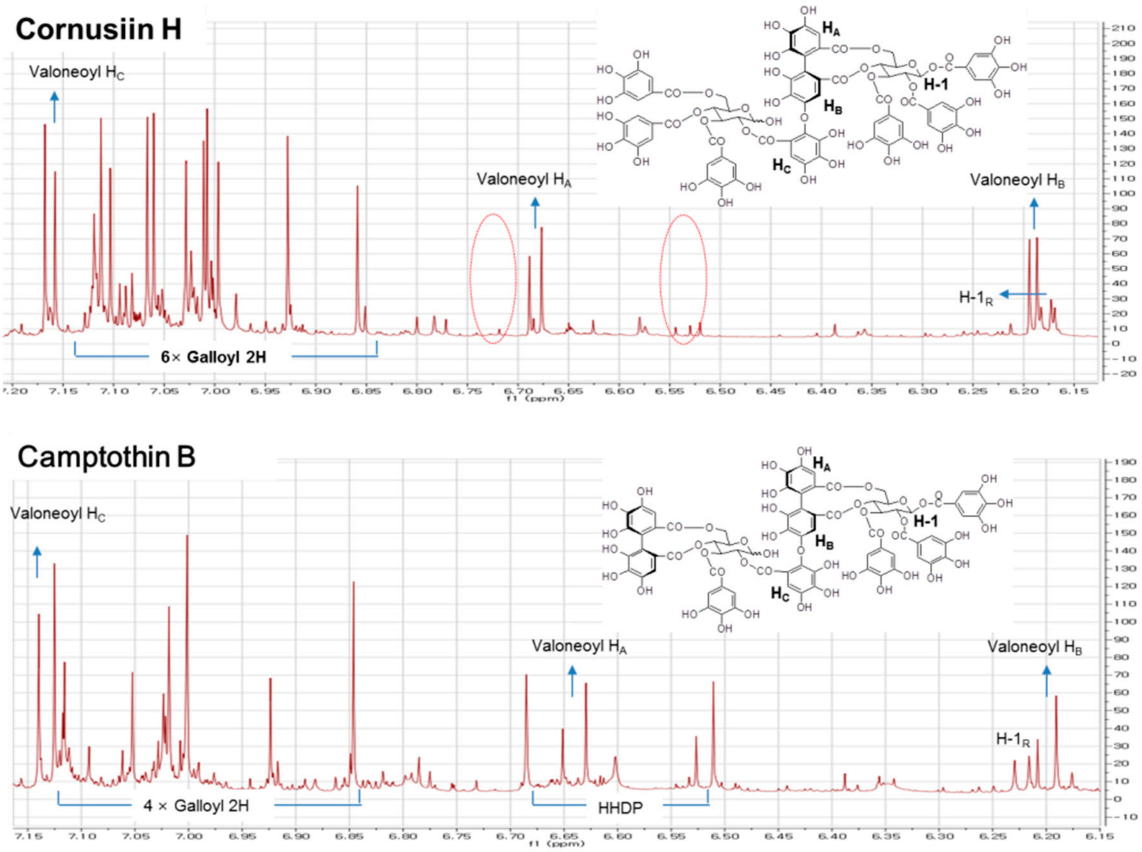

Figure S3. The comparison of aromatic regions of **13** with camptothin B (**12**) on ^{1}H-NMR.

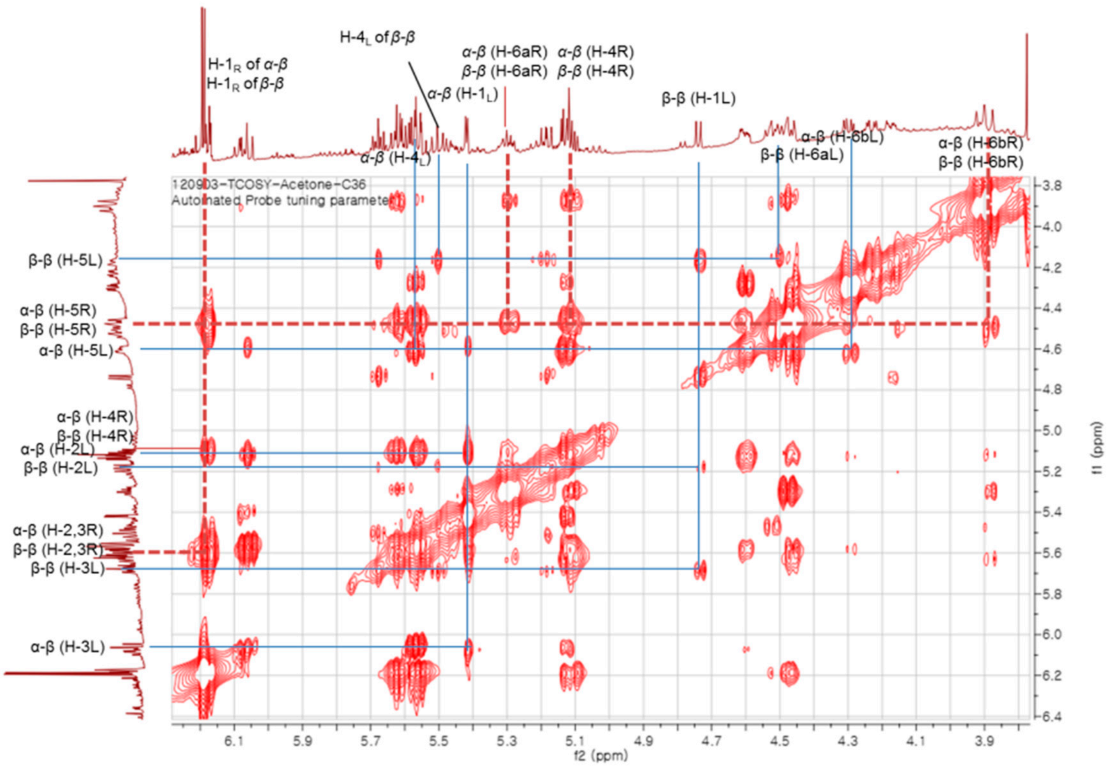

Figure S4. The crucial correlations on the glucose core of **13** in TOCSY. The bold dotted and thin solid lines mean the correlations of the right and left glucose core, respectively.
